# *moc-6*/MOCS2A is necessary for molybdenum cofactor synthesis in *C. elegans*

**DOI:** 10.17912/micropub.biology.000531

**Published:** 2022-02-22

**Authors:** Jennifer Snoozy, Peter C Breen, Gary Ruvkun, Kurt Warnhoff

**Affiliations:** 1 Pediatrics and Rare Diseases Group, Sanford Research, Sioux Falls, SD 57104, USA; 2 Department of Molecular Biology, Massachusetts General Hospital, Boston, MA 02114, USA; 3 Department of Pediatrics, Sanford School of Medicine, University of South Dakota, Sioux Falls, SD 57105 USA

## Abstract

Molybdenum cofactor (Moco) is an essential prosthetic group that mediates the activity of 4 animal oxidases and is required for viability. Humans with mutations in the genes encoding Moco-biosynthetic enzymes suffer from Moco deficiency, a neonatal lethal inborn error of metabolism. *Caenorhabditis elegans *has recently emerged as a useful and tractable genetic discovery engine for Moco biology. Here, we identify and characterize K10D2.7/*moc-6*, the *C. elegans *ortholog of human MOCS2A, a sulfur-carrier protein essential for Moco synthesis. Using CRISPR/Cas9 gene editing, we generate 3 null mutations in K10D2.7/*moc-6 *and with these alleles genetically demonstrate that K10D2.7/*moc-6 *is necessary for endogenous Moco synthesis in *C. elegans.*

**Figure 1. moc-6 is required for C. elegans Moco synthesis:**
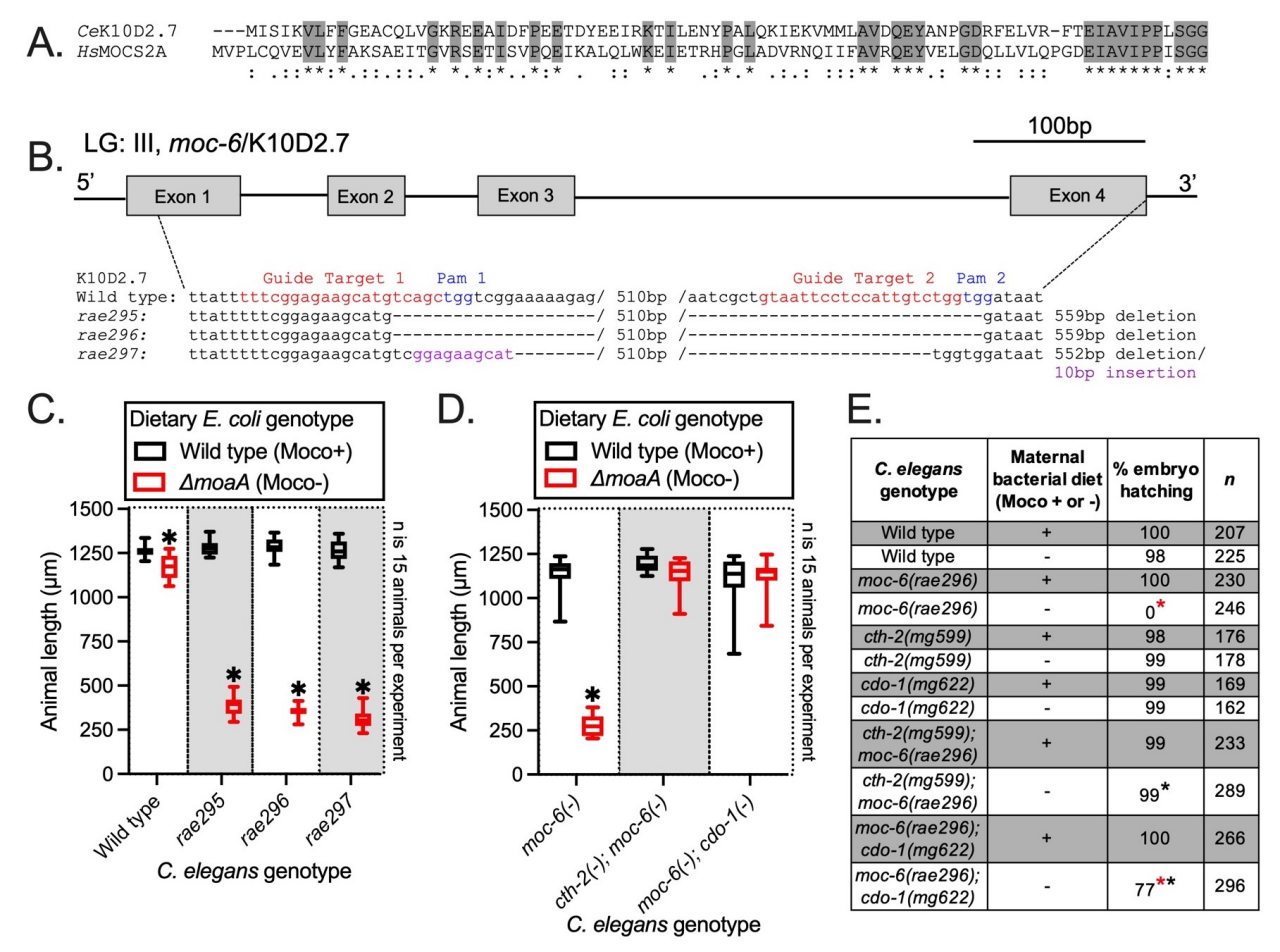
(A) Amino acid alignment between human MOCS2A and *C. elegans* K10D2.7/MOC-6. Shaded amino acids (*) are identical. Strong (:) and weak (.) amino acid similarity are also displayed. (B) Nucleotide sequence for the wild type K10D2.7/*moc-6* locus is displayed as are the sequences for newly generated K10D2.7/*moc-6* deletions *rae295, rae296,* and *rae297.* Guide RNAs were designed targeting the 2 K10D2.7/*moc-6* sequences displayed in red. The 2 corresponding PAM sites are displayed in blue. (C) Wild-type, *rae295, rae296,* and *rae297* animals were cultured from synchronized L1 larvae for 72 hours on wild-type (Moco producing) and *ΔmoaA* mutant (Moco deficient) *E. coli.* Animal length was determined for each condition. (D) *moc-6(rae296), cth-2(mg599); moc-6(rae296),* and *moc-6(rae296); cdo-1(mg622)* mutant animals were cultured from synchronized L1 larvae for 72 hours on wild-type (Moco producing) and *ΔmoaA* mutant (Moco deficient) *E. coli.* Animal length was determined for each condition. For panels C and D, box plots display the median, upper, and lower quartiles, and whiskers indicate minimum and maximum data points. Sample size (*n*) is displayed for each experiment. *, indicates a statistically significant difference (p<0.01, unpaired *t-*test) in *C. elegans* growth on *ΔmoaA E. coli* when compared to corresponding growth on wild-type *E. coli*. (E) Hatching rates for wild-type and mutant *C. elegans* was determined. Moco+ diet indicates that mothers were cultured on wild-type *E. coli* while Moco- indicates that mothers were shifted to *ΔmoaA* mutant *E. coli* at the L4 stage of development, depriving animals of dietary Moco during reproductive adulthood. *n* is the number of embryos scored for hatching for each genotype and condition. * (red), indicates a statistically significant difference (p<0.01, Chi-square test) in the hatching rates of the progeny of *C. elegans* mothers fed *ΔmoaA E. coli* when compared to the corresponding hatching rate when fed wild-type *E. coli*. * (black), indicates a statistically significant difference (p<0.01, Chi-square test) in hatching rates of the progeny of *cth-2(mg599); moc-6(rae296)* and *moc-6(rae296); cdo-1(mg622)* double mutant *C. elegans* mothers fed *ΔmoaA E. coli* when compared to *moc-6(rae296)* single mutant mothers fed *ΔmoaA E. coli*.

## Description

The nematode *C. elegans* has recently emerged as a tractable system for genetic discovery in Moco biology (Warnhoff and Ruvkun 2019; Warnhoff *et al.* 2021). In *C. elegans,* endogenous Moco biosynthesis is not required for growth, development, and reproduction. *C. elegans* mutants defective in Moco biosynthesis are viable because they acquire and use Moco from their bacterial diet (Warnhoff and Ruvkun 2019; Warnhoff *et al.* 2021). Thus, *C. elegans* has 2 redundant pathways for maintaining cellular Moco: endogenous synthesis and dietary acquisition. *C. elegans* is the only known animal for which Moco biosynthesis is dispensable.

The human MOCS2A enzyme is required for Moco synthesis (Schwarz *et al.* 2009). MOCS2A is a small sulfur-carrier protein that is necessary for the conversion of cyclic pyranopterin monophosphate to molybdopterin. This reaction is the second step in the linear synthesis pathway of Moco from guanosine triphosphate (Schwarz *et al.* 2009). Loss-of-function mutations in MOCS2A cause human Moco deficiency, a rare and lethal inborn error of metabolism (Johnson *et al.* 2001). However, the *C. elegans* orthologue of MOCS2A has not been molecularly identified or genetically studied. Using amino acid sequence analysis, we determined that the *C. elegans* K10D2.7 genomic locus encodes aprotein with high amino acid similarity and identity to human MOCS2A (**Fig. 1A**) (Altschul *et al.* 1990). To determine the role K10D2.7 plays in Moco biosynthesis, we used CRISPR/Cas9 to engineer 3 independent deletions of K10D2.7 (**Fig. 1B**) (Cho *et al.* 2013; Ghanta and Mello 2020). These K10D2.7 deletion alleles (*rae295, rae296,* and *rae297*)are likely null mutations because they eliminate >80% of the K10D2.7 coding sequence. As we have shown for other *C. elegans* mutations in Moco biosynthetic machinery*,* the K10D2.7 deletion alleles *rae295, rae296,* or *rae297* cause no defects when animals are cultured under standard laboratory conditions feeding on wild-type *E. coli* which also synthesizes Moco.To determine if K10D2.7 is necessary for endogenous Moco synthesis, we cultured wild-type, *rae295, rae296,* and *rae297* mutant animals on wild-type and *ΔmoaA E. coli,* a mutant bacterial strain that is unable to synthesize Moco. *rae295, rae296,* and *rae297* mutant animals grew well on wild-type (Moco producing) *E. coli* but displayed a completely penetrant developmental arrest when cultured on *ΔmoaA* (Moco deficient) mutant *E. coli* (**Fig. 1C**)*.* In contrast to K10D2.7mutant animals, wild-type *C. elegans* grow well on wild-type or *ΔmoaA* mutant *E. coli,* although we observed a subtle, but statistically significant, reduction in the growth of wild-type animals on *ΔmoaA* mutant *E. coli* (**Fig. 1C**)*.* By eliminating Moco from the diet, we revealed the defect in endogenous Moco synthesis caused by the *rae295, rae296,* and *rae297* mutations. We conclude that K10D2.7 is required for *C. elegans* Moco synthesis and name this gene *moc-6* (MOlybdenum Cofactor biosynthesis).

Moco deficiency in *C. elegans* also results in embryonic lethality when maternal animals are deprived of both endogenous and exogenous sources of Moco during reproductive adulthood (Warnhoff and Ruvkun 2019). *moc-6* mutant *C. elegans* mothers were fed wild-type *E. coli* until the L4 stage of development, one stage prior to reproductive adulthood. These *moc-6* mutant L4 mothers were then shifted onto wild-type or *ΔmoaA* mutant *E. coli* and the hatching rate of their progeny was determined. When *moc-6* mutant animals were shifted to a Moco deficient diet, 0% of their embryos hatched (**Fig. 1E**). In contrast, 100% of *moc-6* mutant embryos hatched when their mothers were fed wild-type *E. coli.* Hatching rates of wild-type embryos were not affected by the maternal diet (**Fig. 1E**). We conclude that maternal dietary Moco is sufficient to support the required Moco-dependent enzymes for embryonic development. Whether dietary Moco is acting maternally or embryonically to support embryonic development remains to be determined.

Previous genetic studies of the *C. elegans* Moco biosynthetic pathwaydemonstrated that Moco is required for the detoxification of sulfites produced by the sulfur amino acid metabolism pathway governed by cystathionase (*cth-2)* and cysteine dioxygenase (*cdo-1*). The activities of *cth-2* and *cdo-1* are required for the developmental arrest that occurs when *C. elegans* are defective in Moco synthesis and lack dietary Moco (Warnhoff and Ruvkun 2019). Loss-of-function/null mutations in *cth-2* or *cdo-1* suppress the developmental arrest displayed by *moc* mutant *C. elegans* grown on Moco-deficient *E. coli* (Warnhoff and Ruvkun 2019). To determine if the developmental arrest of *moc-6* mutant *C. elegans* was also dependent upon *cth-2* and *cdo-1*, we engineered *cth-2; moc-6* and *moc-6; cdo-1* double mutant animals and assessed their growth on wild-type and *ΔmoaA* mutant *E. coli.* In contrast to *moc-6* single mutant animals, *cth-2; moc-6* and *moc-6; cdo-1* double mutant animals grew well on both wild-type and *ΔmoaA* mutant *E. coli* (**Fig. 1D**)*. cth-2* and *cdo-1* single mutant animals grow well on both wild-type and *ΔmoaA* mutant *E. coli* (Warnhoff and Ruvkun 2019)*.* Furthermore, mutations in *cth-2* or *cdo-1* suppressed the embryogenesis defects displayed by *moc-6* mutant mothers fed on Moco-deficient *E. coli* (**Fig. 1E**)*.* Although, we find that *moc-6; cdo-1* double mutant embryos derived from mothers fed *ΔmoaA* mutant *E. coli* do not hatch at the same rate as wild type. Further studies are required to account for this observation. Together, these data demonstrate that *cth-2* and *cdo-1* inhibit normal development when Moco is deficient due to loss of both *moc-6* and dietary Moco. CTH-2 and CDO-1 act in a catabolic pathway to produce sulfite from the sulfur amino acids methionine and cysteine. Sulfites are extremely toxic during Moco deficiency due to inactivity of the Moco-requiring sulfite oxidase enzyme, SUOX-1. Loss of *cth-2* or *cdo-1* prevents the metabolic production of sulfites, alleviates the cellular stress caused by inactive SUOX-1 (concomitant with Moco deficiency), and permits growth, development, and reproduction (Warnhoff and Ruvkun 2019). The mechanism by which sulfite promotes larval arrest and death remains enigmatic in both our *C. elegans* models and in human patients suffering from Moco deficiency.

This study demonstrates that *moc-6* is required for endogenous *C. elegans* Moco biosynthesis and amino acid sequence analyses strongly suggest that *moc-6* is orthologous to human MOCS2A.

## Methods

Animal cultivation:

*C. elegans* strains were cultured using established protocols (Brenner 1974). Briefly, animals were cultured at 20°C on nematode growth media (NGM) seeded with wild-type *E. coli* (BW25113) unless otherwise noted. The wild-type *C. elegans* strain was Bristol N2. All *C. elegans* and *E. coli* strains used in this work are listed in the **Reagents** table.

Engineering deletions of *moc-6/* K10D2.7 using CRISPR/Cas9:

Genome engineering using CRISPR/Cas9 technology was performed using established techniques (Cho *et al.* 2013; Ghanta and Mello 2020). Briefly, 2 guide RNAs were designed and synthesized (IDT) that targeted the K10D2.7 locus (**Fig. 1B**). Cas9 (IDT) guide RNA ribonucleoprotein complexes were directly injected into the *C. elegans* germline (Cho *et al.* 2013). Newly induced deletions were identified in the offspring of injected animals using a PCR-based screening approach. The DNA primers used to screen for new deletions were: 5’-tggtgtagcggcaagtacaa-3’ and 5’-tccctattttgcaccctttg-3’. We were able to isolate and homozygoze 3 independent deletions of K10D2.7; *rae295, rae296,* and *rae297* (**Fig. 1B**)*.*

*C. elegans* growth assays:

*C. elegans* were synchronized at the first stage of larval development (L1). L1 animals were cultured on NGM seeded with wild-type or *ΔmoaA* mutant *E. coli.* Animals were cultured for 72 hours at 20°C and live animals were imaged using an SMZ25 stereomicroscope (Nikon) equipped with an ORCA-Flash4.0 camera (Hamamatsu). Images were captured using NIS-Elements software (Nikon) and processed using ImageJ. Animal length was measured from the tip of the head to the end of the tail. GraphPad Prism software was used to perform statistical tests as well as calculations of median, upper, and lower quartiles.

Quantification of embryo hatching:

To determine the hatching rate of wild-type and mutant *C. elegans*, we performed synchronized egg lays using young adult animals. Embryos were scored for hatching 12-24 hours after being laid. To test the effect of maternal dietary Moco on embryo hatching, mothers were initially grown on wild-type *E. coli* to the L4 stage of development and were shifted onto either wild-type or *ΔmoaA* mutant *E. coli.* Shifted L4 animals developed overnight into young adults on their respective *E. coli* diets and their progeny were assayed for their ability to hatch.

## Reagents

**Table:** 

**Organism**	**Strain**	**Genotype**	**Source**
*C. elegans*	N2	Wild type	CGC
*C. elegans*	USD954	*moc-6(rae295) III*	This work
*C. elegans*	USD955	*moc-6(rae296) III*	This work
*C. elegans*	USD956	*moc-6(rae297) III*	This work
*C. elegans*	GR2257	*cth-2(mg599) II*	CGC
*C. elegans*	GR2260	*cdo-1(mg622) X*	CGC
*C. elegans*	USD959	*moc-6(rae296) III; cdo-1(mg622) X*	This work
*C. elegans*	USD960	*cth-2(mg599) II; moc-6(rae296) III*	This work
*E. coli*	BW25113	Wild type	NIG
*E. coli*	JW0764-2	*ΔmoaA753::kan*	NIG
